# Basement membrane defects in CD151-associated glomerular disease

**DOI:** 10.1007/s00467-022-05447-y

**Published:** 2022-03-12

**Authors:** Richard W. Naylor, Elizabeth Watson, Samantha Williamson, Rebecca Preston, J Bernard Davenport, Nicole Thornton, Martin Lowe, Maggie Williams, Rachel Lennon

**Affiliations:** 1 Wellcome Centre for Cell-Matrix Research, Division of Cell-Matrix Biology and Regenerative Medicine, School of Biological Sciences, Faculty of Biology Medicine and Health, The University of Manchester, Manchester Academic Health Science Centre, Manchester, M13 9PT UK; 2 South West Genomic Laboratory Hub, Bristol Genetics Laboratory, Pathology Sciences, Southmead Hospital, Bristol, UK; 3 Department of Paediatric Nephrology, Royal Manchester Children’s Hospital, Manchester University Hospitals NHS Foundation Trust, Manchester Academic Health Science Centre, Manchester, UK; 4 International Blood Group Reference Laboratory, NHS Blood and Transplant, Filton, Bristol, UK; 5 Division of Molecular and Cellular Function, School of Biological Sciences, Faculty of Biology, Medicine, and Health, Manchester Academic Health Science Centre, University of Manchester, Manchester, UK

**Keywords:** CD151, MER2 Podocyte, Glomerular Basement Membrane, Proteinuria, Kidney disease

## Abstract

**Background:**

CD151 is a cell-surface molecule of the tetraspanin family. Its lateral interaction with laminin-binding integrin ɑ3β1 is important for podocyte adhesion to the glomerular basement membrane (GBM). Deletion of *Cd151* in mice induces glomerular dysfunction, with proteinuria and associated focal glomerulosclerosis, disorganisation of GBM and tubular cystic dilation. Despite this, *CD151* is not routinely screened for in patients with nephrotic-range proteinuria. We aimed to better understand the relevance of *CD151* in human kidney disease.

**Methods:**

Next-generation sequencing (NGS) was used to detect the variant in *CD151*. Electron and light microscopy were used to visualise the filtration barrier in the patient kidney biopsy, and immunoreactivity of patient red blood cells to anti-CD151/MER2 antibodies was performed. Further validation of the *CD151* variant as disease-causing was performed in zebrafish using CRISPR-Cas9.

**Results:**

We report a young child with nail dystrophy and persistent urinary tract infections who was incidentally found to have nephrotic-range proteinuria. Through targeted NGS, a novel, homozygous truncating variant was identified in *CD151*, a gene rarely reported in patients with nephrotic syndrome. Electron microscopy imaging of patient kidney tissue showed thickening of GBM and podocyte effacement. Immunofluorescence of patient kidney tissue demonstrated that CD151 was significantly reduced, and we did not detect immunoreactivity to CD151/MER2 on patient red blood cells. CRISPR-Cas9 depletion of *cd151* in zebrafish caused proteinuria, which was rescued by injection of wild-type *CD151* mRNA, but not *CD151* mRNA containing the variant sequence.

**Conclusions:**

Our results indicate that a novel variant in *CD151* is associated with nephrotic-range proteinuria and microscopic haematuria and provides further evidence for a role of CD151 in glomerular disease. Our work highlights a functional testing pipeline for future analysis of patient genetic variants.

**Graphical abstract:**

A higher resolution version of the Graphical abstract is available as Supplementary information

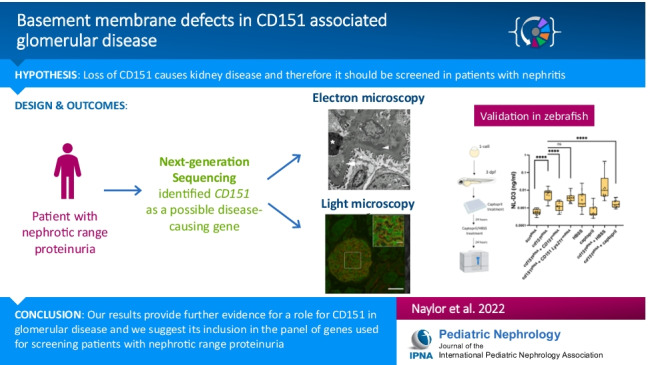

**Supplementary Information:**

The online version contains a graphical abstract available at 10.1007/s00467-022-05447-y.

## Introduction

The glomerulus is responsible for filtration of blood through the multi-layered filtration barrier. This barrier consists of the fenestrated endothelium, the glomerular basement membrane (GBM) and the overlying podocyte layer with interdigitating foot processes. Dysfunction of the filtration barrier can lead to proteinuria, nephrotic syndrome, chronic kidney disease and eventual kidney failure.

Over 80 genes have been associated with inherited nephrotic syndrome, resulting in varying clinical phenotypes [1]. Variants in *CD151* have been implicated in nephrotic syndrome, with loss of function variants in humans associated with kidney failure, bullous skin lesions, nail dystrophy and sensorineural deafness [2].

CD151 is a tetraspanin, part of a large group of transmembrane proteins present in almost all tissues [3], and is critical in podocyte adhesion to the GBM. This cell–matrix interaction is subject to mechanical load during filtration; thus, a robust connection is required to maintain a functional filtration barrier [4]. CD151 interacts with the laminin–integrin–actin axis, specifically via integrin α3β1, increasing the strength of integrin-dependent adhesion of the podocyte to laminin-521 in the GBM [5, 6].

Mouse models of *Cd151* deletion have shown variable phenotypes that appear to be dependent on the genetic background of the mice. Global deletion of *CD151* in mouse models on the FVB background exhibits early, massive proteinuria with associated focal segmental glomerulosclerosis (FSGS) and subsequent kidney failure [7, 8]. Cd151-knockout mice on the C57BL/6 background did not spontaneously develop a kidney phenotype but developed nephrotic range proteinuria with hypertension. This was alleviated with angiotensin-converting enzyme inhibitors, which may act by reducing the mechanical load on the capillary wall by decreasing intraglomerular pressure [9, 10]. The extrarenal features, including sensorineural deafness and bullous skin lesions, have not been replicated in knockout mouse models [7].

We report a patient presentation where a homozygous variant in *CD151* was identified and prompted wider genetic testing in the family, and investigation of kidney biopsy tissue by light and electron microscopy. The findings suggest that this variant in *CD151* is the cause of the disease features observed. To confirm this, we used the zebrafish system to determine the functional significance of the patient *CD151* variant.

## Methods

### Sample preparation for genetics analysis

Genomic DNA was extracted from peripheral blood samples (patients I_1_, I_2_, II_2_) or from saliva samples collected using Oragene kits (DNA Genotek) (patients II_3_, II_4_), using standard extraction methods following manufacturer’s instructions.

### Next-generation sequencing (NGS)

NGS was undertaken for the index patient only. Library preparation was performed using a custom-designed SureSelect (Agilent Technologies) solution-based oligonucleotide target capture assay run on a MiSeq (Illumina Inc). The targeted regions included the coding regions and splicing sites (–20/ + 10 base pairs) of 69 genes associated with steroid-resistant nephrotic syndrome (SRNS) and overlapping clinical indications. A sequencing depth of > 20 × was considered sufficient for variant calling.

### Bioinformatics

Sequence analysis was performed using an open-source in-house pipeline (alignment: BWA; alignment modification and variant calling: GATK; variant annotation: Annovar) with the hg19 human genome as a reference. Variant filtering was performed using a bespoke in-house database GAK with a comparative read-depth approach using ExomeDepth to investigate for copy number variants.

### Variant interpretation and reporting

Variants were classified using the Association of Clinical Genomic Science ACGS (www.acgs.uk.com) best practice guidelines that assimilate the American College of Medical Genetics and genomics recommendations [11] for NHS use; only pathogenic, likely pathogenic and variants of uncertain clinical significance appropriate for clinical follow-up were reported.

### Familial testing

Subsequent familial variant testing in relatives of the index patient was performed using standard Sanger sequencing and a primer pair designed for *CD151* (NM_004357.4) exon 8.

### Human kidney biopsy

Kidney biopsy sections from the index, female patient at age 4 years and control kidney tissue (non-affected tissue of tumour nephrectomy) were used with Regional Ethics Committee approval 06/Q1406/38 including parental consent for research and publication.

### Blood group serology

Control red blood cells (RBCs), one example of human anti-MER2 (anti-MER2 1) and one example of mouse monoclonal anti-MER2 (anti-MER2 2) typing sera, were from the IBGRL reference collection. For the human anti-MER2, serological testing was carried out by standard low-ionic strength saline solution (Lorne Laboratories Ltd.) tube indirect antiglobulin test (IAT) with polyclonal anti-human globulin reagent (Millipore). For the mouse monoclonal anti-MER2, serological testing was carried out by IAT in ID-PNH Test cards (BioRad). Agglutination was scored on a scale of 0 (negative) to 4 + (strongest positive).

### Immunofluorescence

Control and patient biopsy tissue samples were used. Sections were dewaxed to water using a Leica ST5010 Autostainer. Antigen retrieval was performed first by heat-induced antigen retrieval in acidic citrate buffer (pH 2.0) for 20 min, followed by incubation at room temperature in acidic urea/glycine buffer (pH 3.2) for 30 min. Samples were washed in PBS then blocked in 1% donkey serum, 2% BSA, and 0.1% Triton X-100 in PBS for 45 min at room temperature. Primary antibodies (rat anti-collagen IV α3; 1:100; Chondrex 7076 and rabbit anti-CD151; 1:100; Abcam ab125363) were diluted in blocking buffer and incubated overnight at 4˚C in a humidified chamber. Secondary antibodies (Alexa488 anti-rabbit; 1:200; Invitrogen; A21206 and Alexa594 anti-rat; 1:200; Invitrogen; A21209) were diluted in blocking buffer and incubated on slides for 45 min at room temperature in a humidified chamber. After washing, slides were dried overnight and mounted with ProLongTM Diamond Antifade Mountant (ThermoFisher; P36961).

Images were collected on a Zeiss Axioimager.D2 upright microscope using a 20 × / 0.50 EC Plan-neofluar and 40 × / 0.75 Plan-neofluar objective and captured using a Coolsnap HQ2 camera (Photometrics) through Micromanager software v1.4.23. Specific band-pass filter sets for DAPI, FITC and Texas red were used to prevent bleed through from one channel to the next. Images were then processed and analysed using Fiji/ImageJ. Statistical analysis was performed using GraphPad Prism version 8.4.3 for Windows, GraphPad Software, San Diego, California USA, (www.graphpad.com).

### Transmission electron microscopy image analysis

Transmission electron microscopy (TEM) images acquired in the clinical pathology laboratory were analysed. Distances were measured in Fiji/ImageJ using a grid method; 124 measurements were taken and normalised to the length of the GBM. The mean ± SD was calculated using GraphPad Prism version 9 for Windows, GraphPad Software, San Diego, California USA (www.graphpad.com).

### Zebrafish husbandry and manipulations

Zebrafish were maintained and staged according to established protocols [12] and in accordance with the personal project license of Professor Rachel Lennon (P1AE9A736) and under the current guidelines of the UK Animals Act 1986. Embryos were collected from group-wise matings of *nphs2:egfp* or *NL-D3* reporter fish [13]. Progeny from these crossings were injected with *cd151* crRNAs whose design was based on the algorithm described by Wu et al. [14]. The 4 crRNAs (in 5’–3’ orientation) were:

- cd151 crRNA 1 – CACAGCGCCACCTGCCAACT
- cd151 crRNA 2 – GGGCTGCTGCGCCACTATCA
- cd151 crRNA 3 – TTTAGCTTACGTCTATTATC
- cd151 crRNA 4 – TGCAGCAGCTGTCGGGCACC
- scr crRNA 1 – CAGGCAAAGAATCCCTGCC
- scr crRNA 2 – TACAGTGGACCTCGGTGTC
- scr crRNA 3 – CTTCATACAATAGACGATG
- scr crRNA 4 – TCGTTTTGCAGTAGGATCG

crRNAs (and tracrRNA) were diluted to a stock concentration of 20 µM in RNAse-free water. For the injection mix, crRNA and tracrRNA were combined at a 1:1 ratio to make the gRNA (final concentration of each gRNA was 4 µM). To anneal the crRNA and tracrRNA, these components were placed in a PCR machine running the following program: 5 min at 95˚C, ramp down to 85˚C at –2˚C/sec, ramp down to 25˚C at –0.1C/sec. Once combined, Cas9 (NEB #M0646) was added to the mixture at a final concentration of 4 µM along with 1 × Cas9 buffer. 5 nl of this injection mixture was then injected into the cytoplasm of 1-cell stage *NL-D3* transgenic embryos. These crispant embryos were maintained at 24 hours post-fertilisation (hpf) and grown to 3 days post-fertilisation (dpf) before being processed in the proteinuria assay system (see below). 5 dpf crispants were also placed in 1 ml of TRIzol and homogenised by sonication. RNA extraction was performed using the Purelink RNA Extraction kit (Thermo #12183020), and cDNA synthesis was performed using Takara PrimeScript cDNA synthesis kit (#6111A).

### RT-PCR

For RT-PCR, cDNA was diluted to 1 ng/µl and mixed with 7.8 µM of primers and an equal volume of 2 × SYBRGreen (#4309155). The primers used are described below:

- actb F – TCACCACCACAGCCGAAAG
- actb R – AGAGGCAGCGGTTCCCAT
- nphs1 F – AGTCACCACTGCTGATGCG
- nphs1 R – TGCTGGTGTTCCCTTTCAGG
- col4a3 F – AACTTGTCGCTACGCCTCTC
- col4a3 R – ATTGCCTCGCATACGGAACA
- col4a4 F – CTGGCTTTAAGGGACCTCCG
- col4a4 R – AAGCAGACTGTAGCCGTTCC
- lama5 F – TGGAGGGACCCAAATGCAAG
- lama5 R – ACCAGAACCCGAGGCTGTAT

The PCR was run on a Bio-Rad CFX96 Touch Real-Time PCR machine. All analyses of the data used the ∆∆Ct method of quantification. RT-PCRs were run in triplicate with -RT and -cDNA controls.

### Zebrafish proteinuria reporter and drug treatments

Using the *NL-D3* transgenic line, crispant and control fish that were treated with captopril were grown to 3 dpf and three embryos were placed in a single well of a 96-well dish. Embryo media (E3) was removed and replaced with 200 µl of E3 containing 10 µM captopril (Bio-Techne, #4455/50) or 0.1% DMSO as a vehicle control. These embryos were left for 24 h, and the same treatment was performed again at 4 dpf. After another 24 h, 50 µl of E3 media was isolated and placed into 50 µl of the Nano-Glo^Ⓡ^ Luciferase reporter system (Promega, #N1120) in a fresh opaque 96-well plate. Luminescence intensity was determined on a FlexStation 3 multi-mode microplate reader. Relative luminescence units (RLUs) were converted to NL-D3 amounts (ng/ml) using a standard curve (see [13]). For Hank’s Balanced Salt Solution (HBSS) treatments, embryos were injected with 5 nl of neat HBSS (Sigma, #H9394) into the pericardial cavity at 4 dpf and then processed for proteinuria analysis in the same way as described above. The effects of HBSS on glomerular capillary width were determined in the *nphs2:egfp* line using a Leica stereofluorescence microscope. For rescue experiments, *cd151* crispant embryos were injected with 50 ng/μl of either 5’ capped mRNA encoding wild-type human *CD151* or variant Lys211* CD151. Full-length human *CD151* and Lys211* CD151 open-reading frames were obtained from IDT as gblocks and subcloned into the pCS2 + overexpression vector. mRNA synthesis was carried out using the SP6 mMessage Machine kit (ThermoFisher #AM1340).

## Results

### Clinical presentation

**Fig. 1:**
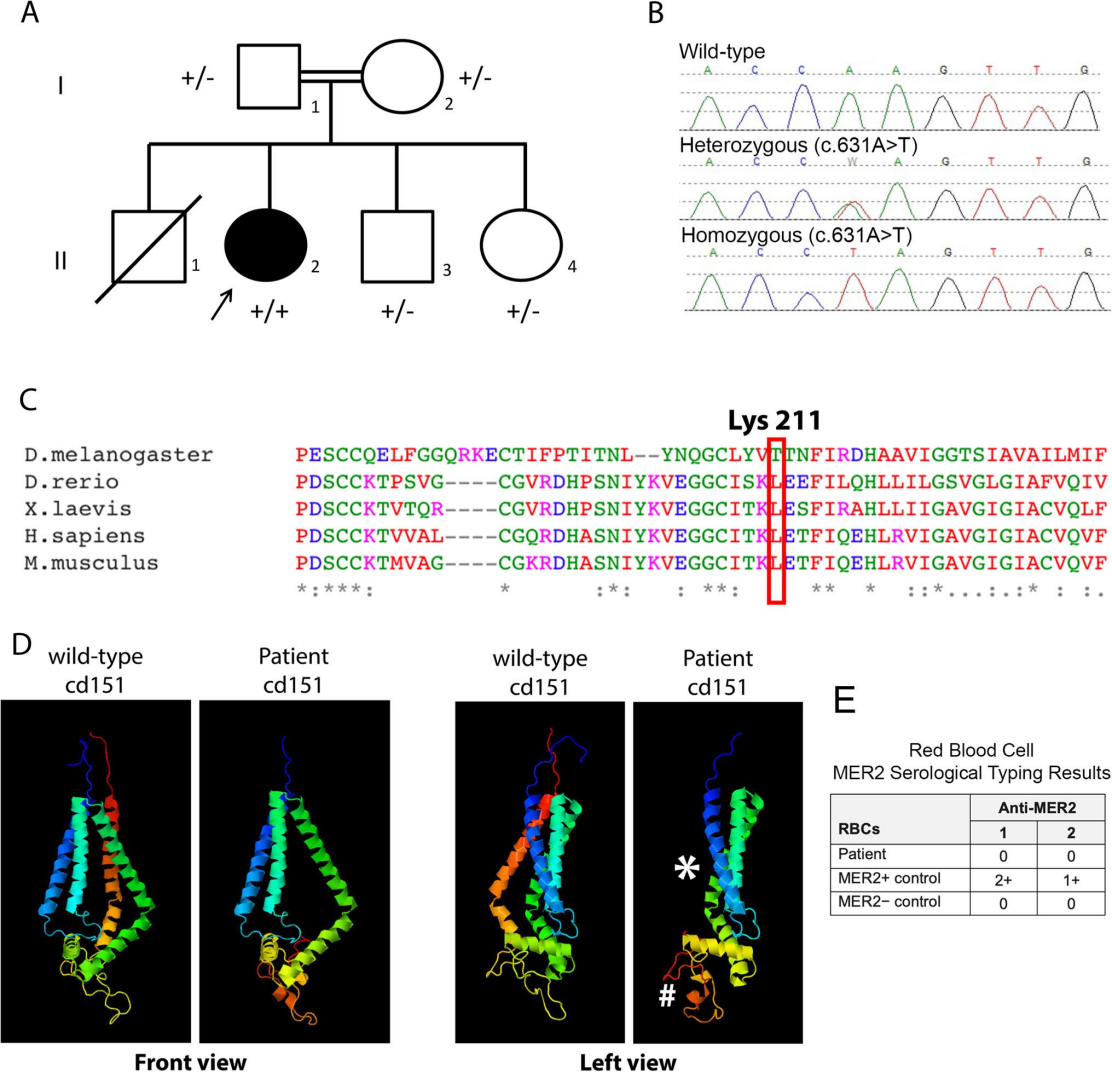
Pedigree, genetic sequence, conservation, protein structure. A) Pedigree of a family affected with nephropathy, nail dysplasia and skin lesions and a *CD151* variant. Solid symbols represent affected individuals. The index patient is marked with an arrow.  +/- denotes a heterozygote, + / + denotes a homozygous pathogenic variant. B) Chromatograms of DNA Sanger sequencing reads from affected individuals. DNA was available for testing for all individuals except II_1_. C) The variant introduces a premature stop codon at Lysine 211. Here, conservation of this lysine is shown across various species. D) Structural modelling of the Lys211* variant effect on CD151 protein using the Phyre2 web portal for protein modelling [15]. Asterisk shows position of missing C-terminal transmembrane helix, hashtag highlights the EC2 loop domain that has grossly changed structure when compared to the wild-type CD151 protein. E) Table showing results of blood serology typing. The patient’s RBCs were found to be negative with two examples of anti-MER2, thereby indicating the MER2-negative phenotype

We report the index presentation of a 2-year-old female who presented with recurrent febrile urinary tract infections despite appropriate antibiotic prophylaxis. Other than nail dystrophy and hypopigmented skin lesions, there were no dysmorphic features present and antenatal, post-natal and past medical history were uneventful. Systemic antifungal treatment was also given initially as the nail dystrophy was thought to be due to infection. Parents were first cousins. An older sibling, with ear and cardiac abnormalities, sadly died aged 7 days from neonatal sepsis. There are two younger siblings, who are well, and following screening were not found to have proteinuria or urinary tract infections (Fig. 1A). However, sibling II_4_ was diagnosed with bilateral grade III microtia with canal atresia and conductive hearing loss. There was no family history of kidney disease.

An initial ultrasound revealed kidneys of normal size. A possible duplex system was demonstrated on the left side with the suggestion of upper pole scarring. Additionally, intermittent dilatation of the kidney pelvis was noted on the left, with mild dilatation of the left distal ureter. A subsequent ultrasound, approximately 12 months later, demonstrated kidneys of normal size and shape, although increased echogenicity of both kidneys was observed. There was no evidence of hydronephrosis, and the pelvis and ureteric dilatation initially noted on the left side had resolved. A cystogram subsequently demonstrated a structurally normal bladder with no evidence of vesico-ureteric reflux. During cystoscopy, inflammatory changes were noted in the bladder, likely secondary to recurrent urinary tract infections. Urine microscopy consistently revealed proteinuria and microscopic haematuria. Subsequent calculation of urinary protein creatinine ratio demonstrated nephrotic-range proteinuria (ranging from 400 to 1200 mg/mmol). Thus, enalapril was commenced. Excretory kidney function was within the normal range and blood pressure remained within acceptable limits for height and weight. There was no clinical evidence of oedema, and the serum albumin remained within the normal range.

In view of nephrotic-range proteinuria, a kidney biopsy was undertaken which revealed striking GBM defects. Specifically, the biopsy demonstrated areas of increased thickness with laminations and focal wrinkling of the GBM around mesangial regions. There were areas of mesangial increase, and electron-dense structures were identified in the para-mesangial and subepithelial space. Other than a subcapsular focus of sclerosed glomeruli with interstitial fibrosis, there was no evidence of FSGS and the podocyte foot processes around the capillary loops were preserved. Overall, the GBM changes identified were unusual and were not diagnostic of any one condition. Thus, nephrotic-range proteinuria and histological evidence of glomerular disease prompted genetic panel testing for proteinuria.

Targeted NGS of the index case with a panel of 69 genes associated with SRNS and overlapping indications revealed a novel homozygous truncating variant in *CD15*1 (NM_004357.4) c.631A > T p.(Lys211*) (Fig. 1B). No other variants of clinical significance were identified. The presence of the *CD151* c.631A > T p.(Lys211*) variant in exon 8 was confirmed by Sanger sequencing and subsequent familial testing for this variant demonstrated both parents to be heterozygous, confirming homozygosity in the index patient. Both unaffected siblings of the index case were also heterozygous. Using ACMG/ACGS scoring criteria, the variant was classified as a variant of uncertain significance in a candidate gene with application of the codes PM2 and PP4 at a moderate level given the biopsy functional data and PM3 at a supporting level of strength.

This missense variant changes a conserved lysine residue (Fig. 1C) to a stop codon, which entirely deletes the final transmembrane domain of CD151 and disrupts the extracellular EC2 domain that is important for interactions with integrin α3β1 (Fig. 1D). Given this, the CD151 Lys211* variant is predicted to have a loss of function. Interestingly, there are two reports in the literature supporting loss of function as a mechanism of disease for this gene [2, 16]. Homozygous frameshift and splice site variants in *CD151* were associated with epidermolysis bullosa, nail dystrophy and proteinuria leading to kidney failure. RBCs of patients in Crew et al. [2] were found to be negative when tested with antibodies to the Raph blood group system antigen (MER2), which is a component of the CD151 protein. We therefore examined agglutination as a readout of the immunoreactivity of the patient red blood cells (RBCs) against sera (human and mouse) with anti-MER2 antibodies. We also included MER2^+^ and MER2^–^ RBCs as controls. We found no reactivity with the patient RBCs or the negative control RBCs, but we did find reactivity to the positive control RBCs (Fig. 1E). This finding is consistent with the absence of CD151/MER2 on patient RBCs.

### Kidney biopsy reveals basement membrane defects

**Fig. 2:**
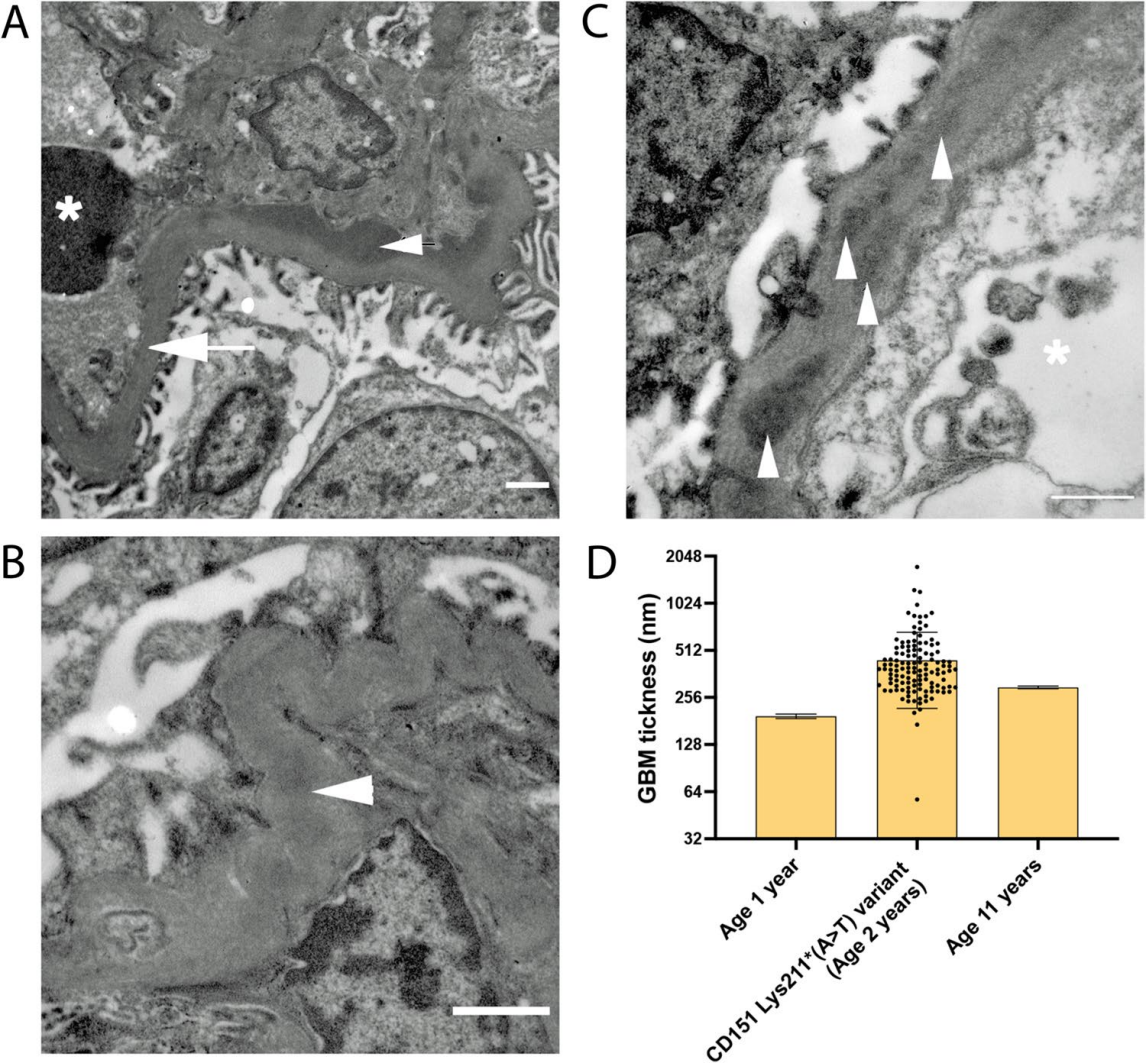
TEM images showing changes in GBM in patient with loss of function variant in the *CD151* gene. A) Thickened region of GBM (arrow) opposite to the capillary lumen containing an erythrocyte (asterisk). Electron dense deposits can be seen in the GBM para-mesangial region (arrowhead). **B)** Wrinkling of GBM around mesangial regions was observed along with electron-dense deposits (arrowhead). **C)** Smaller electron dense deposits (arrowheads) within the subepithelial region of the GBM (asterisk: capillary lumen). Scale bars = 1 µm. **D)** quantification of patient GBM thickness based on TEM images of the case patient kidney biopsy and previously published measurements of GBM thickness in a one- and 11-year-old extrapolated from Ramage et al. (2002). Measurements were taken in Fiji/ImageJ, and mean ± SD was calculated in GraphPad Prism

As described above, ultrastructural analysis by TEM identified significant disruption in the glomerular filtration barrier in the patient. Electron dense deposits were prominent throughout the GBM (Fig. 2A–C, arrowheads) and the GBM appeared wrinkled in long stretches (as shown in Fig. 2B). Overall, the GBM appeared thicker than expected for the age of the patient from which the biopsy was taken. Quantification of GBM thickness in the TEM images showed that it was on average 442.9 土 225.2 nm (Fig. 2D). Ramage et al. (2002), using a formula to measure the true harmonic mean thickness, determined the average GBM thickness at one year of age to be 194 土 6.5 nm [17]. The GBM becomes thicker with age, measuring 297 土 6 nm at 11 years of age (Fig. 2D) [17]. The thickness of the patient biopsy sample (taken at 2 years of age) is therefore ~ 2.3-fold thicker than a healthy 1-year-old GBM and ~ 1.49-fold thicker than a healthy 11-year-old GBM (Fig. 2D). No signs of foot-process effacement were observed. In summary, the analysis of TEM images highlights significant alteration in the glomerular filtration barrier, with impact on GBM thickness and ultrastructure.

**Fig. 3:**
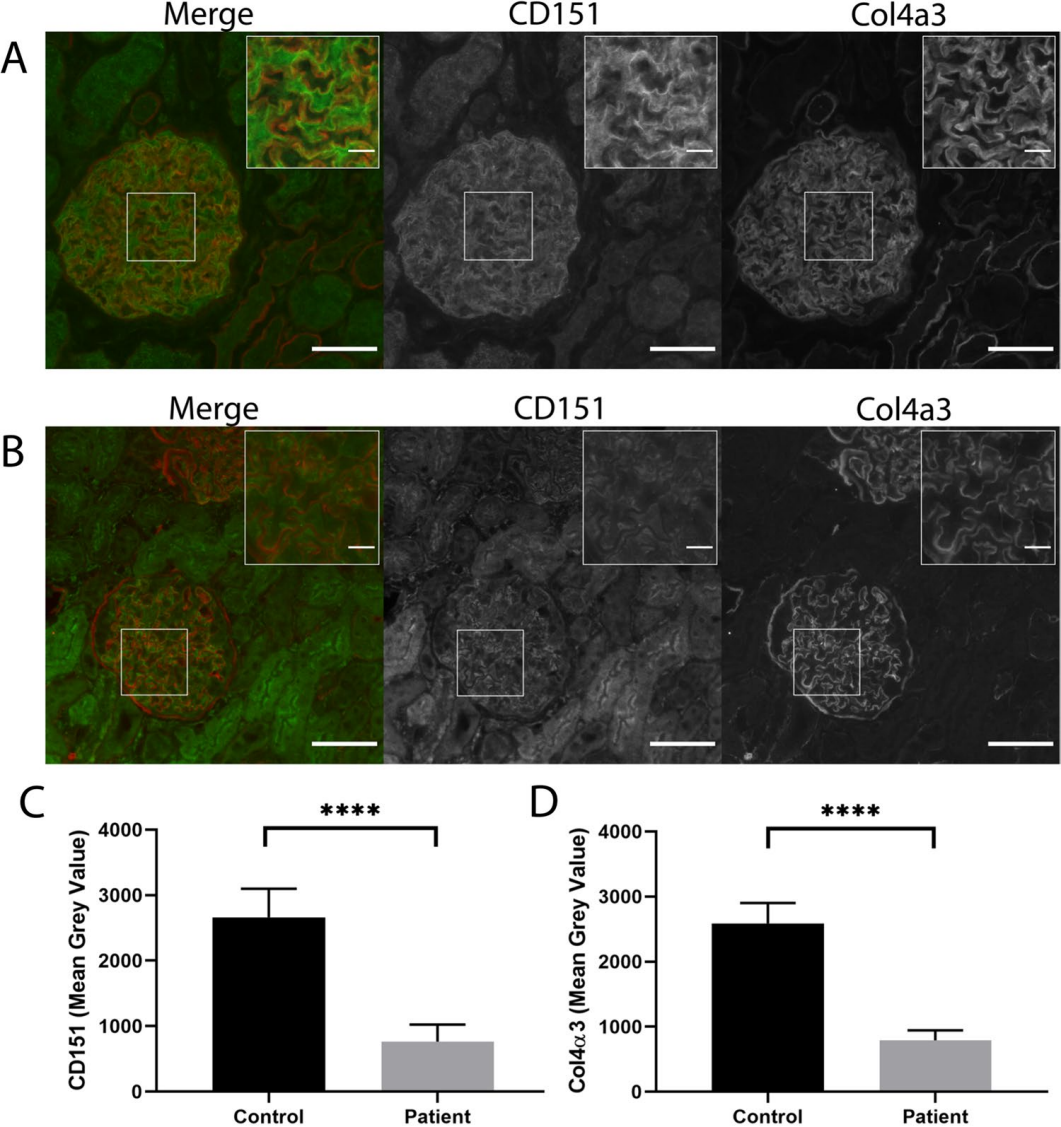
Immunofluorescence images of CD151 and Col4α3 in normal human kidney glomeruli and a patient with loss of function variant in the *CD151* gene. IF images left to right: co-IF (CD151 green; Col4α3 red), CD151 and Col4α3. Scale bars = 50 µm, insert scale bar = 10 µm. **A)** Representative glomeruli from normal human adult control; **B)** representative glomeruli from patient kidney biopsy – fluorescence was significantly less than control and was adjusted using Fiji/ImageJ to make easily visible; **C)** quantification of CD151 expression in control and patient glomeruli; **D)** quantification of Col4α3 expression in control and patient glomeruli. Mean grey values were obtained for the region of the capillary tuft using Fiji/ImageJ and the mean ± SD and an unpaired t-test (*p* < 0.0001) was performed using GraphPad Prism. Control glomeruli n = 26; Patient glomeruli *n*  = 17

We also performed CD151 immunofluorescence on healthy adult kidney tissue (Fig. 3A) and patient kidney biopsy tissue (Fig. 3B) to visualise the spatial distribution of CD151. We observed that CD151 was significantly reduced in the glomeruli of patient kidney biopsy tissue relative to the normal adult glomeruli (quantified in Fig. 3C). In addition to CD151, we examined *COL4A3*, which encodes type IV collagen ɑ3, which is specifically expressed by podocytes and is present in the GBM as part of a collagen IV heterotrimer. Type IV collagen ɑ3 chains were detected in the normal adult tissue (Fig. 3A), but fluorescence intensity was lower in the patient biopsy (Fig. 3B, quantification in Fig. 3D). These results suggest that loss of CD151 causes a reduction in other GBM components, which might also exacerbate the proteinuria phenotype in the case patient. We note, however, that our inability to compare age-matched control glomeruli might account for some of the difference in fluorescence intensity observed.

### Functional variant analysis in the zebrafish

**Fig. 4:**
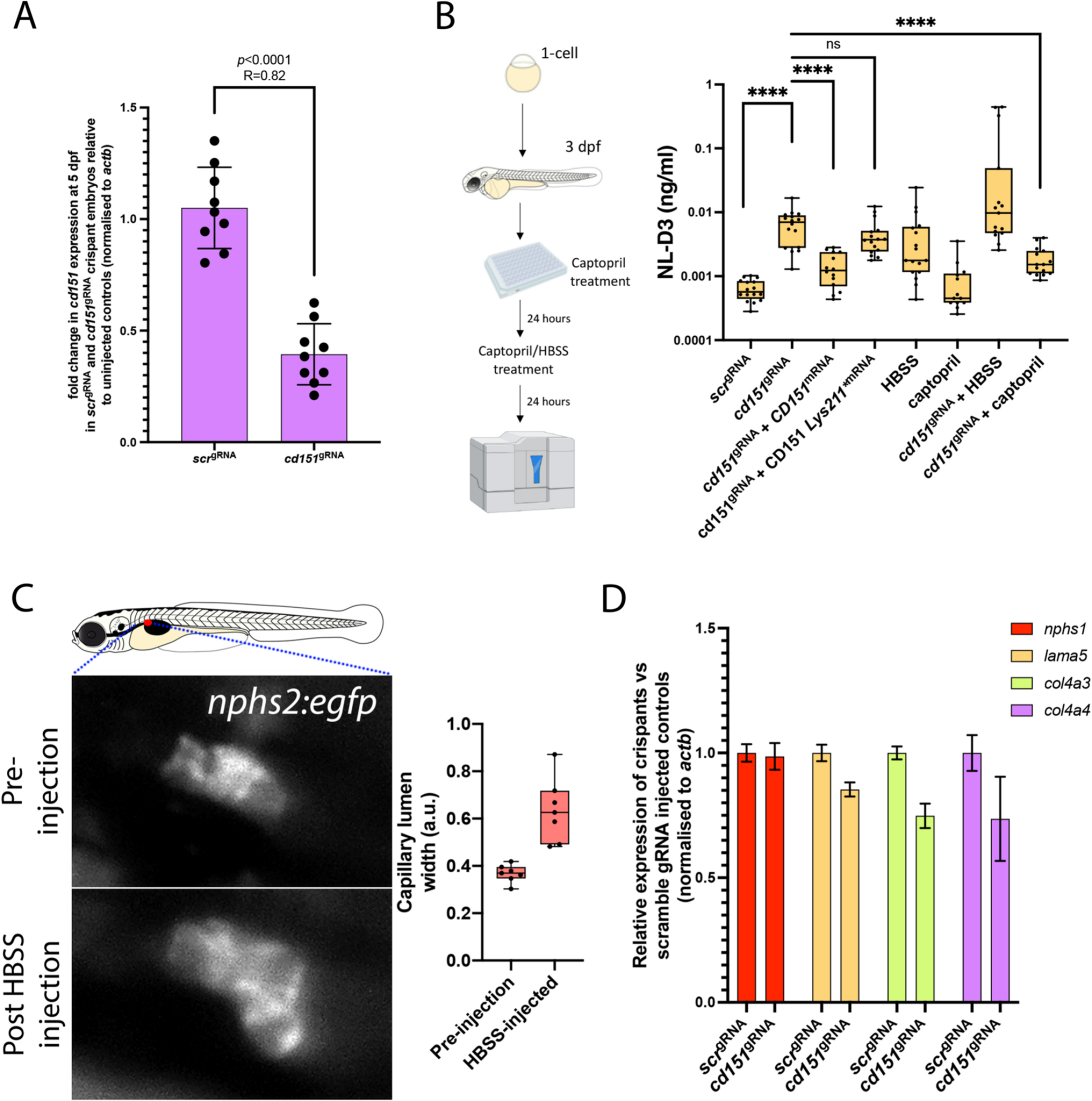
Depletion of *cd151* in zebrafish recapitulates human phenotypes. A) Histogram shows RT-PCR for scrambled gRNA-injected controls and *cd151* gRNA-injected crispants. Each data point is a biological replicate (*n* = 9), and relative expression is to un-injected wild-type controls. All Ct values were normalised to *actb* expression. **B**) Schematic on left shows experimental set-up for zebrafish proteinuria analysis. Histogram shows the levels of proteinuria (in relative luminescence units (RLUs)) of the indicated treatments. **C**) Top panel shows a normal glomerulus in a 3 dpf *nphs2:egfp* zebrafish embryo. Bottom panel shows the same embryo with a dilated glomerulus 1 h after HBSS injection into the cardiac cavity. Histogram illustrates the measurements of capillary width in the glomeruli of embryos pre- and post-HBSS injection. **D**) Histogram of RT-PCR results showing the relative gene expression of the indicated genes in *cd151* crispant embryos compared to stage-matched controls. All gene expression data were normalised to *actb*

We next aimed to functionally test the role of the CD151 Lys211* variant in glomerular function by characterising the effects of depleting cd151 (the ortholog of human CD151) in zebrafish and rescuing phenotypes with CD151 mRNA containing wild-type and variant sequence. The zebrafish is an excellent tool for studying glomerular development and disease as it shares structural and molecular homology with human glomeruli [18, 19]. *cd151* is strongly expressed in the glomerulus of zebrafish embryos from 2 days post fertilisation (dpf) [20]. Given this, we used a CRISPR-Cas9 approach [14], which generates knockout-like phenotypes in F_0_ crispants, to deplete cd151 in the zebrafish. gRNAs were injected into one-cell stage embryos and RT-PCR analysis showed robust knockdown of transcript levels when analysed at 5 dpf (*n* = 9, Fig. 4A). Knockdown was performed in our zebrafish proteinuria *NL-D3* reporter (as described in [13]), which uses a nanoluciferase luminosity assay to quantify protein excreted into the embryo media (Fig. 4B). Analysis of proteinuria in these crispant embryos between 4 dpf and 5 dpf showed that depletion of *cd151* caused proteinuria, increasing the amount of NL-D3 present in the embryo media ~ 10.3-fold (Fig. 4B). Co-injection of wild-type *CD151* mRNA and *cd151* gRNAs partially rescued this proteinuria. Whilst these fish had proteinuria, the level was reduced to a ~ 2.2-fold increase (Fig. 4B). No rescue of the *cd151* crispant proteinuria phenotype was achieved with CD151 Lys211* mRNA, with proteinuria non-significantly different to *cd151* crispants (Fig. 4B). These results demonstrate the specificity of targeting to cd151 that our gRNAs achieved, and they confirm that the CD151 Lys211* variant is functionally inactive and leads to proteinuria.

To test whether the reduction of cd151 in zebrafish affected the integrity of the glomerular filtration barrier and its ability to withstand intraglomerular mechanical loads, we used captopril to lower, and Hank’s Balanced Salt Solution (HBSS) to increase systemic blood pressure. Captopril is an angiotensin-converting enzyme inhibitor that has been shown to lower blood pressure in zebrafish via inhibition of the renin–angiotensin system [21]. Treatment of control embryos showed no effect on glomerular function (Fig. 4B). However, in *cd151* crispants, captopril reduced proteinuria ~ 7.5-fold, which almost completely rescued the proteinuria phenotype as levels of NL-D3 were only ~ 1.3-fold higher compared to controls (Fig. 4B). These results therefore support the concept that cd151 functions to provide greater mechanical strength to the glomerular filtration barrier. Injection of HBSS into the cardiac cavity increased glomerular capillary width (Fig. 4C) and induced proteinuria in both control embryos and *cd151* crispants (Fig. 4B). These changes suggest that this treatment increases systemic blood pressure. Given that *cd151* depletion further exacerbated proteinuria when systemic blood pressure increased, we conclude that in the zebrafish, cd151 supports the glomerular response to heightened blood pressures and mechanical loads in a similar manner to as has been shown in mice [9].

Given that immunofluorescence of type IV collagen ɑ3 was reduced in our patient biopsy, we also analysed zebrafish *cd151* crispants to determine the effect of cd151 loss on expression of glomerular genes. We found that *nphs1* expression was unchanged and for genes that encode for components of the GBM, *lama5* was slightly reduced and *col4a3* and *col4a4* were reduced by ~ 25% (Fig. 4D). These results suggest loss of cd151 function has an impact on the expression of key GBM components. This is consistent with the lowering of type IV collagen ɑ3 expression in the patient biopsy analysis (Fig. 3) and may contribute to the continuing decline in kidney function observed in patients.

## Discussion

Here, we outline the clinical presentation of a young girl with kidney disease associated with novel truncating variant in the *CD151* gene. We find that CD151 protein abundance is significantly reduced in the biopsy of the case patient and ultrastructural analysis by TEM identified thickening, lamination, and wrinkling of the GBM. Immunofluorescence showed that the patient biopsy also had reduced levels of type IV collagen ɑ3, an integral component of the GBM that is required for proper filtration.

Our findings provide further insight into the role of CD151 in human health, in particular the role of CD151 in maintaining podocyte adhesion and providing mechanical strength to the glomerular filtration barrier. The initial presentation of nail dystrophy (thickening) and recurrent urinary tract infections in the index case resulted in the patient being given systemic antifungal treatments. This highlights a lack of information available to clinicians regarding diagnosing CD151 disease-associated variants. CD151 is also highly expressed in the bladder urothelium in the Human Protein Atlas; therefore, further investigation is required to determine whether there is a connection between the functional role of CD151 in the bladder and susceptibility to lower urinary tract infections observed in the index patient. In TEM images, we observed large electron dense deposits in the GBM, which were also reported by Crew et al. [2]. These deposits resemble immune-like deposits observed in other kidney diseases associated with nephrotic-range proteinuria, such as IgA nephropathy and membranous nephropathy. However, we were unable to identify what these electron-dense regions contain. We also observed limited podocyte foot-process effacement, which might be due to the early life-stage at which the patient biopsy was analysed. It has recently been proposed that GBM compression by interdigitating podocytes is important for determining pore size [22, 23]. Thus, we speculate that the cause of the nephrotic-range proteinuria in the index case is a consequence of reduced podocyte adhesion to the underlying GBM matrix, leading to a reduced capability to compress the GBM and regulate size selectivity in the filter.

Our secondary structure analysis of wild-type and Lys211* CD151 identified the truncated variant protein to be entirely missing the C-terminal cytoplasmic domain and the fourth transmembrane domain. A large extracellular loop (the EC2 domain) exists between the third and fourth transmembrane domains and is the integrin α3β1 interaction domain [24]. The EC2 domain spans from Leu149 to Glu213, and thus, the Lys211* variant also truncates this domain by three amino acid residues. Predictive secondary structure analysis showed the EC2 domain to be grossly altered, suggesting it is unable to properly function in binding to integrin receptors. However, it is likely that the premature stop codon will lead to nonsense-mediated mRNA decay [25], preventing transcripts from this variant being translated. In support of this, we were unable to detect CD151 protein by immunostaining using a polyclonal antibody targeting amino acids 154–203. Crew et al. [2] reported three cases where a frameshift variant causing a loss of the EC2 domain resulted in kidney failure and nail dystrophy as observed in our study. They also reported the patients had additional symptoms, including pretibial bullous skin lesions, neurosensory deafness, and bilateral lacrimal duct stenosis [2]. These additional disease features suggest phenotypic variation might be specific to the different variants described, or due to other genes driving or modifying the phenotype. We suggest that genetic modifiers are more likely given the mutant proteins in both our study and Crew et al. [2] are unlikely to generate translated protein that reaches the plasma membrane. It is also possible that other disease-causing variants in our index case are present and so further investigation is required to conclusively prove that the CD151 Lys211* mutation is the cause of the phenotypes observed in the patient.

To provide more evidence, we verified our findings in the zebrafish system. A major issue for genetic variant interpretation is the lack of a pipeline to reliably test the disease associations of detected variants in a controlled research environment. To overcome this constraint, we depleted cd151 in zebrafish and attempted to rescue the proteinuria disease phenotype using human *CD151* mRNA-containing wild-type or Lys211* sequence. We found that wild-type transcripts rescued the phenotypes, but the Lys211* variant transcripts did not. Taken together, these results confirm the patient variant in CD151 is functionally deficient and highlights the use of our zebrafish proteinuria reporter system as a tool for functional analysis of variants associated with glomerular diseases.

In conclusion, our work describes functional testing of the impact of a *CD151* variant on protein function. Our findings highlight the importance of CD151 to glomerular health and aid the curation of gene panels for disease-causing variants. This study provides further evidence that *CD151* causes glomerular disease, and we therefore propose that *CD151* is included in the panel of genes for investigating glomerular proteinuria.

## Supplementary Information

Below is the link to the electronic supplementary material.

Graphical Abstract 5447 (PPTX 163 KB)
