# Supplementary material for: Basement membrane defects in CD151-associated glomerular disease
Source: Pediatr Nephrol. 2022 Mar 12;37(12):3105–15. doi: 10.1007/s00467-022-05447-y (PMC9587066; doi:10.1007/s00467-022-05447-y)
Supplement: Supplementary file 1 — Graphical Abstract 5447 (PPTX 163 KB) [file 467_2022_5447_MOESM1_ESM.pptx]

## Slide 1
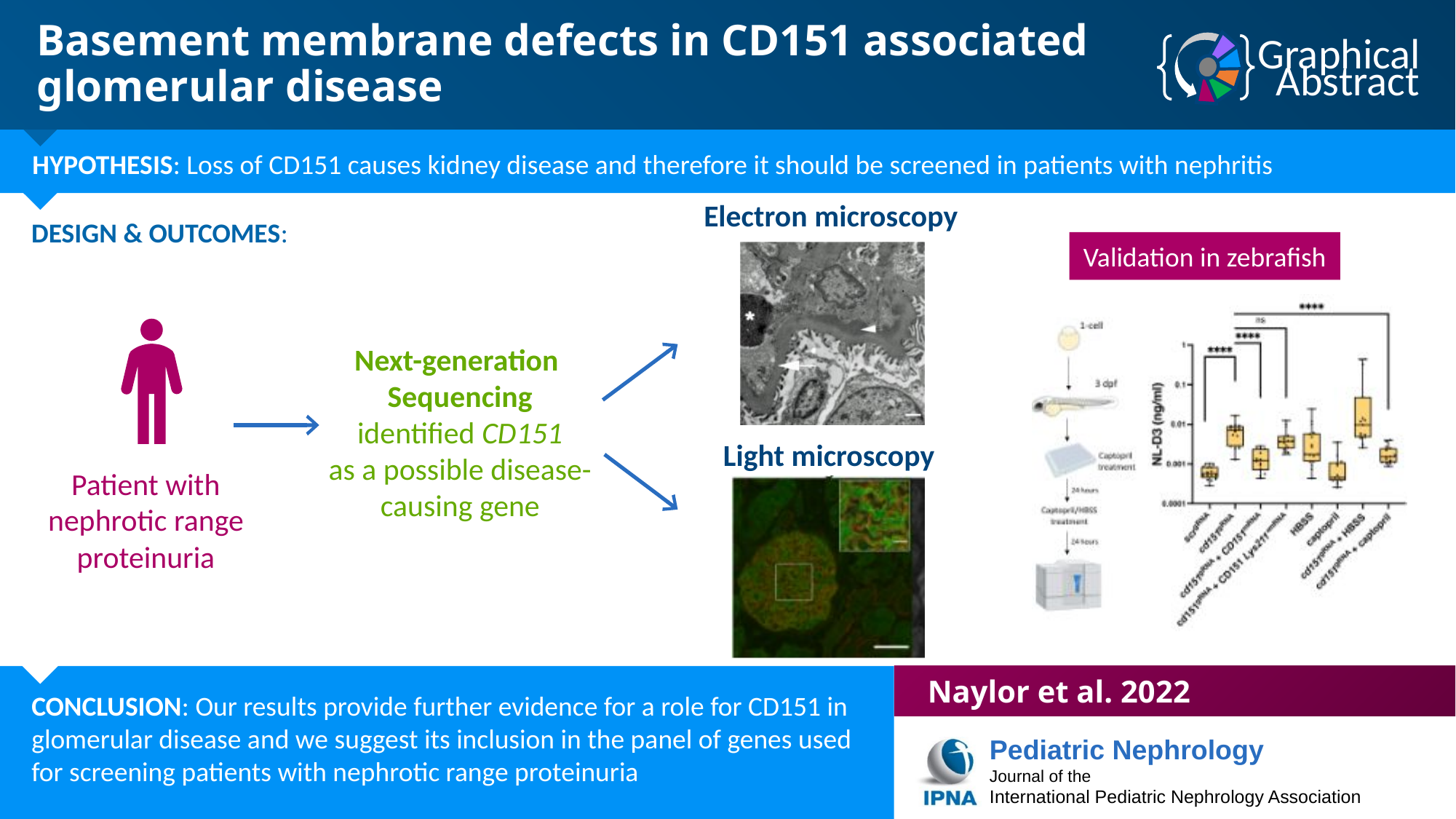

Basement membrane defects in CD151 associated
glomerular disease
HYPOTHESIS: Loss of CD151 causes kidney disease and therefore it should be screened in patients with nephritis
Electron microscopy
DESIGN & OUTCOMES:
Validation in zebrafish
Next-generation
Sequencing
identified CD151
as a possible disease-
causing gene
Light microscopy
Patient with nephrotic range proteinuria
Naylor et al. 2022
CONCLUSION: Our results provide further evidence for a role for CD151 in glomerular disease and we suggest its inclusion in the panel of genes used for screening patients with nephrotic range proteinuria
